# Supplementary material for: Regulation of the luminescence mechanism of two-dimensional tin halide perovskites
Source: Nat Commun. 2022 Jan 10;13:60. doi: 10.1038/s41467-021-27663-0 (PMC8748536; doi:10.1038/s41467-021-27663-0)
Supplement: Supplementary file 1 — Supplementary Information [file 41467_2021_27663_MOESM1_ESM.pdf]

Supplementary Information

Regulation of the Luminescence Mechanism of Two-Dimensional Tin Halide Perovskites

Tianju Zhang[1,2], Chaocheng Zhou[3,4], Xuezhen Feng[5], Ningning Dong[1,2], Hong Chen[5], Xianfeng Chen[4,6], Long Zhang[2], Jia Lin[3]* and Jun Wang[1,2,7]*

1. Laboratory of Micro-Nano Optoelectronic Materials and Devices, Shanghai Institute of Optics and Fine Mechanics, Chinese Academy of Sciences, Shanghai 201800, China.
2. Center of Materials Science and Optoelectronic Engineering, University of Chinese Academy of Sciences, Beijing 100049, China.
3. Department of Physics, Shanghai Key Laboratory of Materials Protection and Advanced Materials in Electric Power, Shanghai University of Electric Power, Shanghai 200090, China.
4. State Key Laboratory of Advanced Optical Communication Systems and Networks, School of Physics and Astronomy, Shanghai Jiao Tong University, Shanghai 200240, China
5. State Environmental Protection Key Laboratory of Integrated Surface Water-Groundwater Pollution Control, Guangdong Provincial Key Laboratory of Soil and Groundwater Pollution Control, School of Environmental Science and Engineering, Southern University of Science and Technology, Shenzhen 518055, China.
6. Collaborative Innovation Center of Light Manipulation and Applications, Shandong Normal University, Jinan 250358, China
7. CAS Center for Excellence in Ultra-intense Laser Science, Shanghai 201800, China

*E-mail: jwang@siom.ac.cn; jlin@shiep.edu.cn

Supplementary Figure 1. The top-view scanning electron microscopy (SEM) images. 1
Supplementary Figure 2. High-resolution XPS spectra of the Sn $3d_{3/2}$ and Sn $3d_{5/2}$ regions in the 2D Sn-based perovskite films with different organic cations. 2
Supplementary Figure 3. UPS spectra of the 2D Sn-based perovskite films with different organic cations. 3
Supplementary Figure 4. Pump energy-dependent TA spectra of $(PEA)_2SnI_4$. 4
Supplementary Figure 5. Pump energy-dependent TA spectra of $(PEA)_2SnI_4$ within 5ps. 5
Supplementary Figure 6. Methods of analyzing bandgaps for $(PEA)_2SnI_4$, $(BA)_2SnI_4$, $(HA)_2SnI_4$, and $(OA)_2SnI_4$ perovskites 6
Supplementary Figure 7. Energy band diagram calculated by density functional theory (DFT) calculations. 7
Supplementary Figure 8. The power-law dependence of the integrated PL intensity of perovskites. 8
Supplementary Figure 9. Elliott theory of excitonic absorption of the four perovskites: 9
Supplementary Figure 10. Temperature-dependent PL of different perovskite films. 10
Supplementary Figure 11. The analysis of the influence of temperature on the PL of the $(PEA)_2SnI_4$ samples. 11
Supplementary Figure 12. PL spectra and their Gaussian fit of four perovskites at 77 K. 12
Supplementary Figure 13. Steady-state Raman spectra of the four perovskites ($(PEA)_2SnI_4$, $(BA)_2SnI_4$, $(HA)_2SnI_4$, and $(OA)_2SnI_4$) 13
Supplementary Figure 14. Evolution-associated spectra obtained upon global analysis of the TA data of the four perovskites 14
Supplementary Figure 15. Contour map of the temperature dependence of the TA spectra of the four samples with the delay time of 0.5 ps. 15
Supplementary Figure 16. Transient dynamic characteristics of the $(PEA)_2SnI_4$ perovskite. . 16
Supplementary Figure 17. Characteristics of time-resolved PL of $(PEA)_2SnI_4$. 17
Supplementary Figure 18. The GSB relaxation kinetics of the $(PEA)_2SnI_4$ sample. 18
Supplementary Figure 19. Temperature-dependent normalized GSB relaxation kinetics of the four samples. 19
Supplementary Figure 20. Schematic diagram of the TA system. 20
Supplementary Table 1. 21
Supplementary Table 2. 23
Supplementary Table 3. 24
Supplementary Note 1. Analysis of SCXRD data of 2D perovskites 25
Supplementary Note 2. UPS analysis of the different perovskites 27
Supplementary Note 3. Analysis of the transition process of the PB2 bleaching peak at 520nm 28
Supplementary Note 4. Fitting of absorption spectra based on Elliott's formula 30
Supplementary Note 5. The analysis of temperature-dependent PL 31
Supplementary Note 6. exciton-phonon coupling model 33
Supplementary Note 7. Band filling and dynamic Burstein–Moss shift 35
Supplementary Note 8. Pump fluence dependence of TA lifetime 36
Supplementary References 37

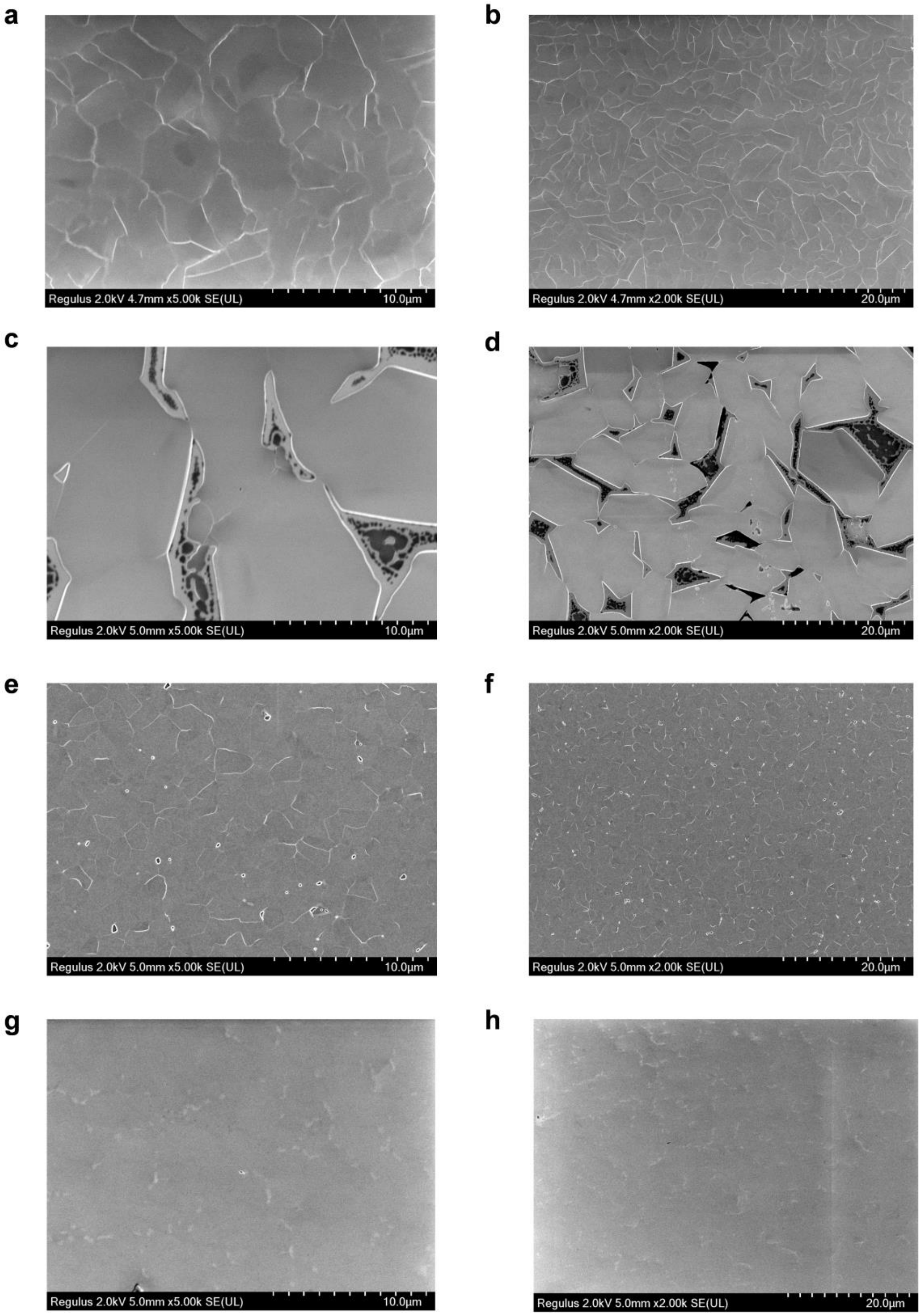

Supplementary Figure 1. The top-view scanning electron microscopy (SEM) images. (a) and (b) $(PEA)_2SnI_4$, (c) and (d) $(BA)_2SnI_4$, (e) and (f) $(HA)_2SnI_4$, and (g) and (h) $(OA)_2SnI_4$ thin films.

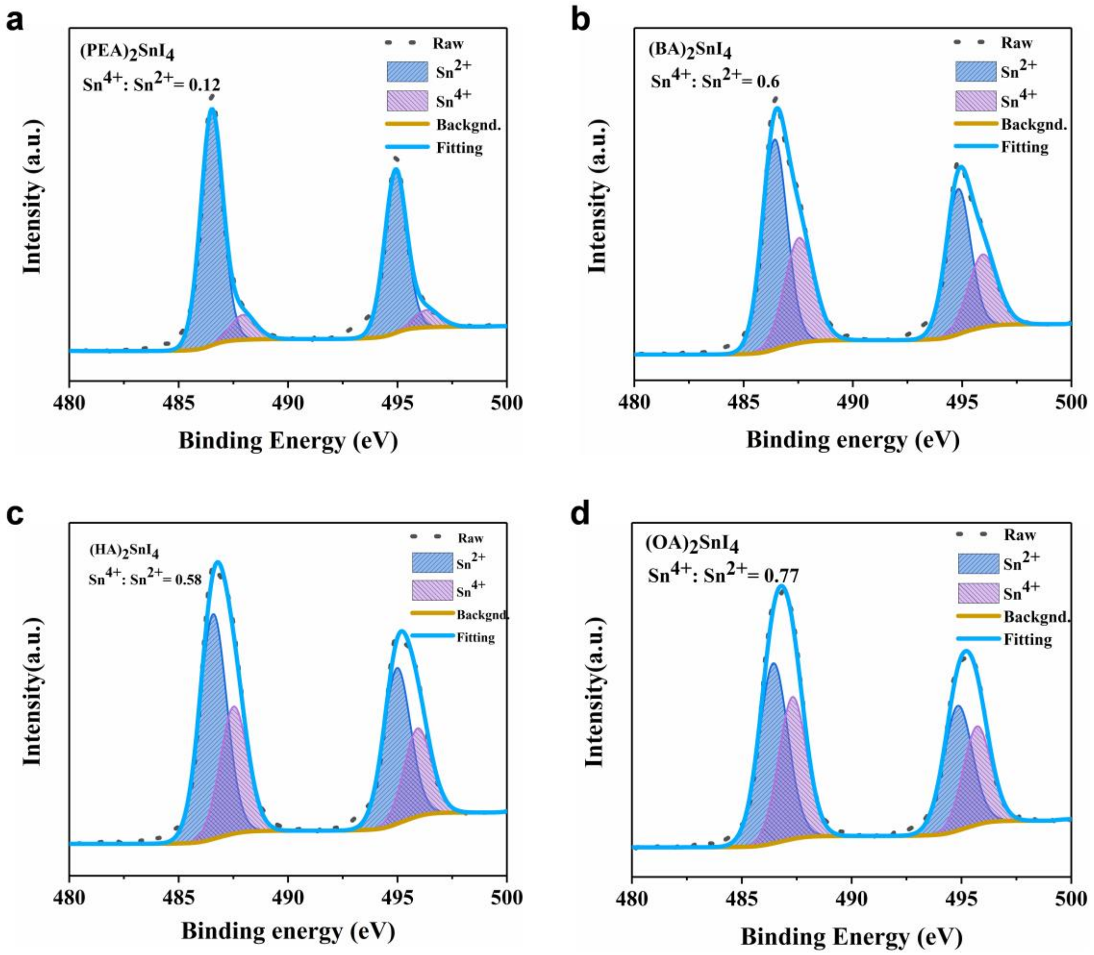

Supplementary Figure 2. High-resolution XPS spectra of the Sn 3$d_{3/2}$ and Sn 3$d_{5/2}$ regions in the 2D Sn-based perovskite films with different organic cations. (a) $(PEA)_2SnI_4$, (b) $(BA)_2SnI_4$, (c) $(HA)_2SnI_4$, and (d) $(OA)_2SnI_4$. The regions corresponding to only the Sn^{2+} (486.6 and 495.1 eV) and the Sn^{4+} (487.4 and 488.9 eV) compounds are represented by blue and red shadows, respectively. The black dots represent the experimental data, the yellow line represents the experimental baseline, and the turquoise solid lines represent the overall results of the two Gaussian distributions.

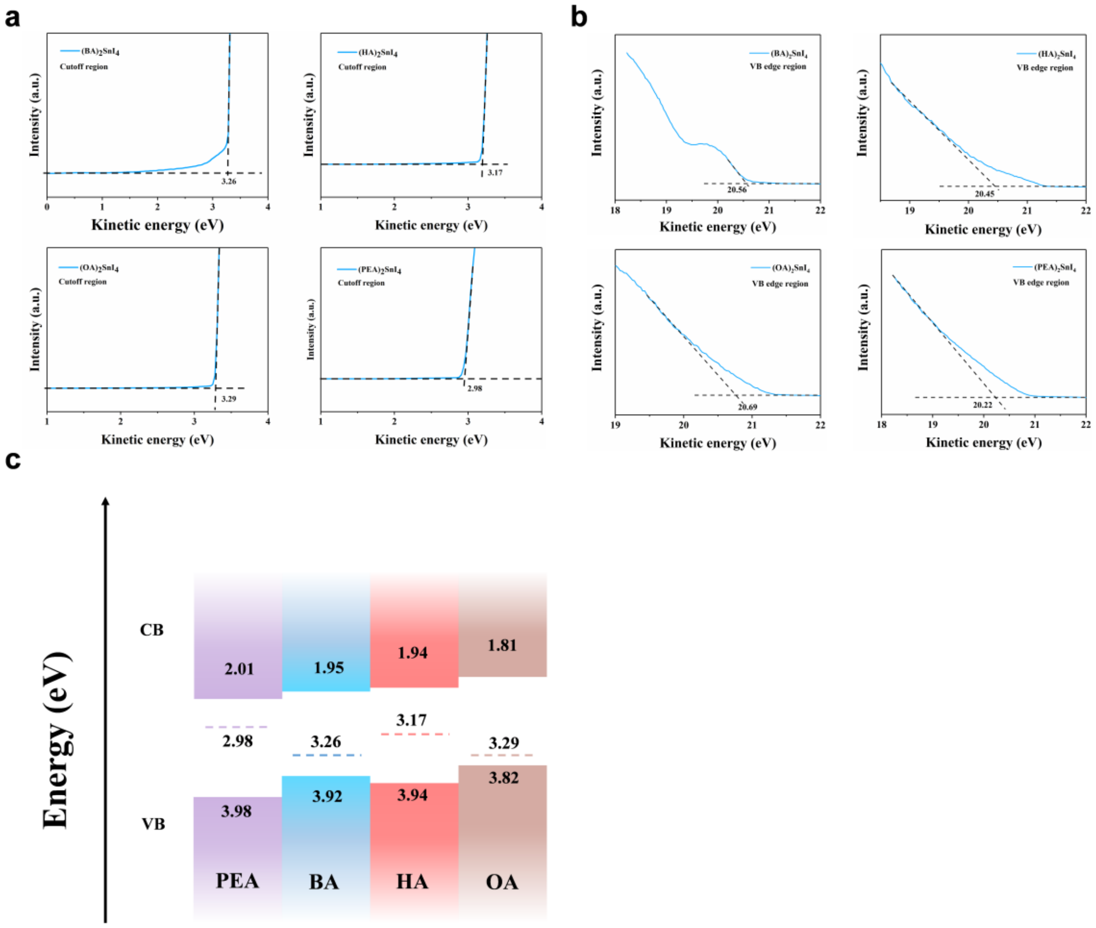

Supplementary Figure 3. UPS spectra of the 2D Sn-based perovskite films with different organic cations. (a) The cutoff region, (b) VB edge region, and (c) the derived energy band diagram of the different perovskite films.

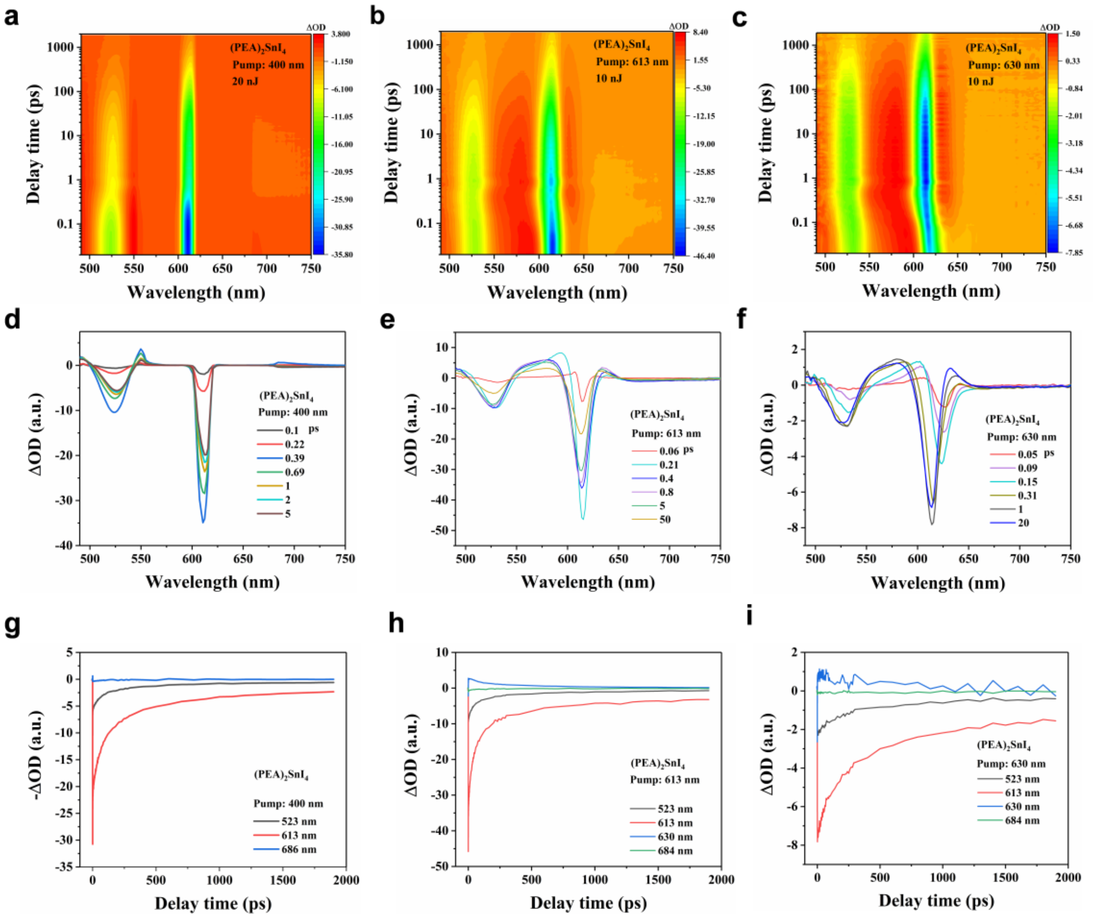

Supplementary Figure 4. Pump energy-dependent TA spectra of $(PEA)_2SnI_4$. 2D pseudocolor TA spectra obtained by pumping at a wavelength of (a) 400 nm, (b) 613 nm, and (c) 630 nm. TA spectra at different delay times with pumping at (d) 400 nm, (e) 613 nm, and (f) 630 nm. Relaxation kinetics at different wavelengths with pumping at (g) 400 nm, (h) 613 nm, and (i) 630 nm.

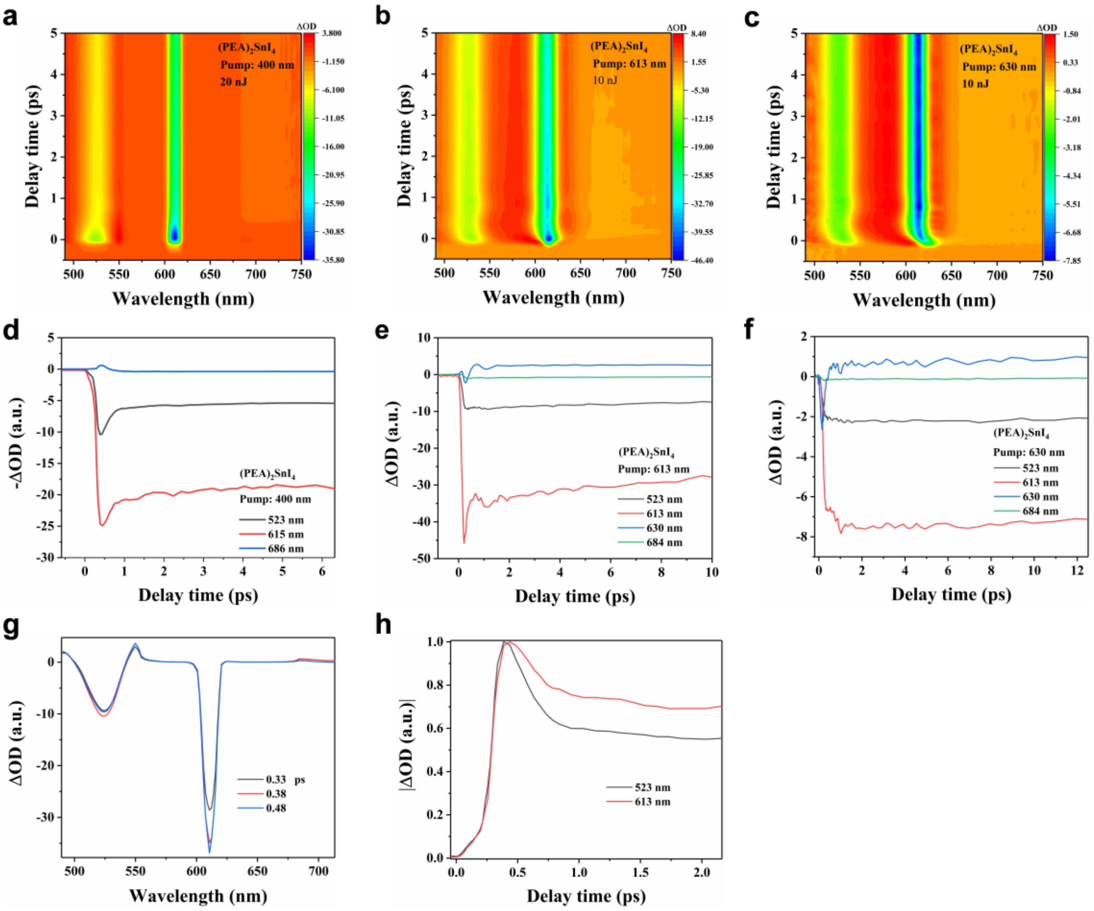

Supplementary Figure 5. Pump energy-dependent TA spectra of $(PEA)_2SnI_4$ within 5ps. 2D pseudocolor TA spectra obtained by pumping at a wavelength of (a) 400 nm, (b) 613 nm, and (c) 630 nm. Relaxation kinetics at different wavelengths with pumping at (d) 400 nm, (e) 613 nm, and (f) 630 nm. (g)TA spectra at different delay times by pumping at a wavelength of 400 nm. (h) Relaxation kinetics at different wavelengths by pumping at a wavelength of 400 nm.

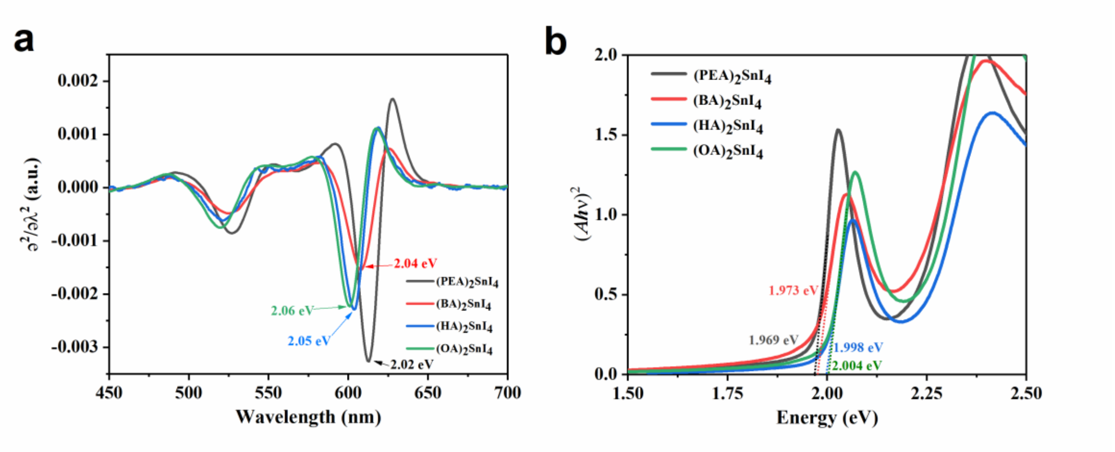

Supplementary Figure 6. Methods of analyzing bandgaps for $(PEA)_2SnI_4$, $(BA)_2SnI_4$, $(HA)_2SnI_4$, and $(OA)_2SnI_4$ perovskites. (a) The second-derivative analysis of the linear absorption spectra and (b) Tauc plot method.

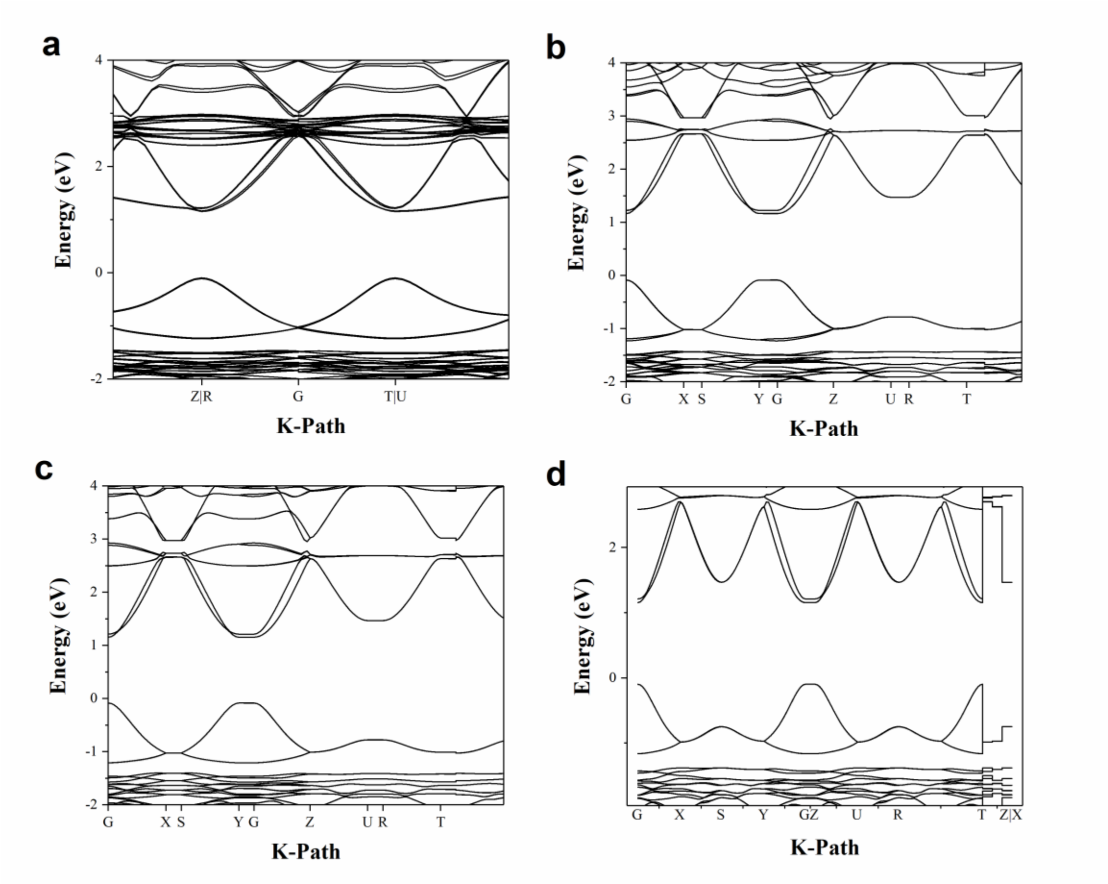

Supplementary Figure 7. Energy band diagram calculated by density functional theory (DFT) calculations. (a) Band Gap of $(PAE)_2SnI_4$ is 1.53 eV. (b) Band Gap of $(BA)_2SnI_4$ is 1.852 eV. (c) Band Gap of $(HA)_2SnI_4$ is 1.998 eV. (d) Band Gap of (OA)2SnI4 is 2.01 eV.

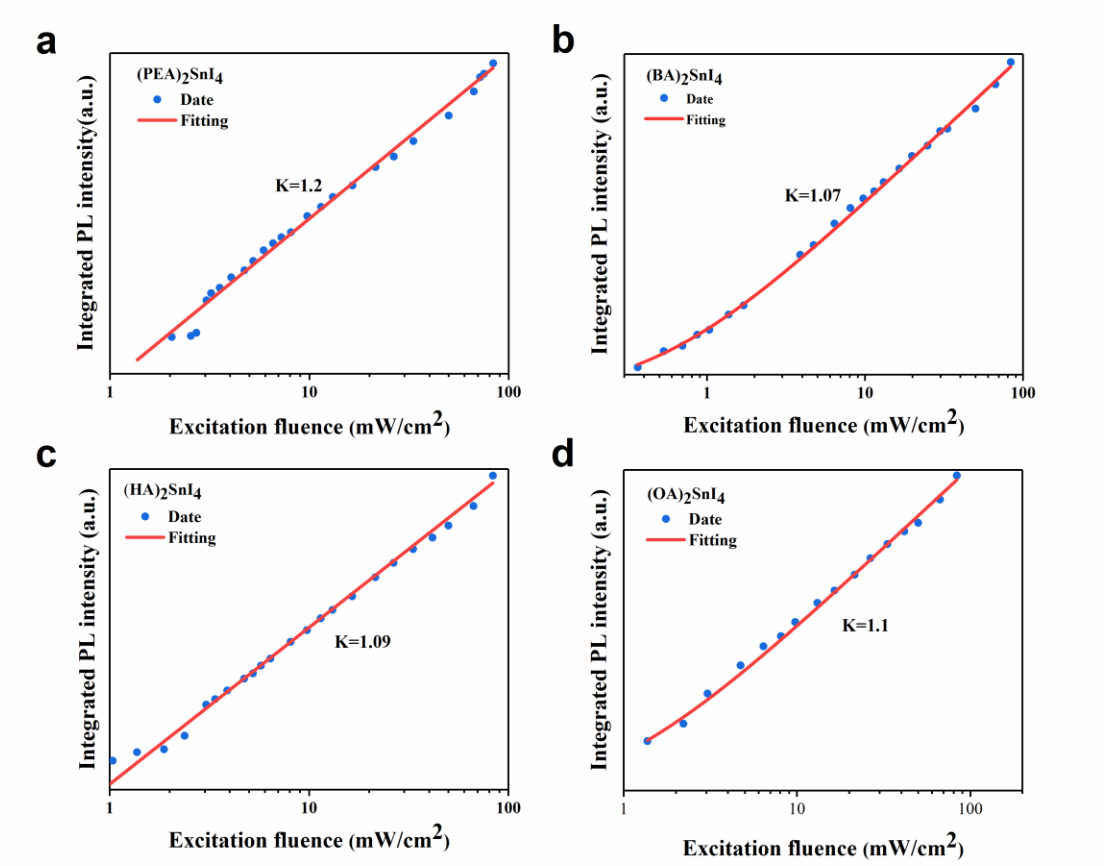

Supplementary Figure 8. The power-law dependence of the integrated PL intensity of perovskites. Logarithmic relationship between the integrated PL intensity I_{PL} and excitation density I_{ex}. The integrated PL intensity is a power-law function of the excitation density, that is $I^{PL} \sim I_{ex}{}^{K}$. The power-law dependence K is (a) 1.2 for $(PEA)_2SnI_4$, (b) 1.07 for $(BA)_2SnI_4$, (c) 1.09 for $(HA)_2SnI_4$ and (d) 1.1 for $(OA)_2SnI_4$.

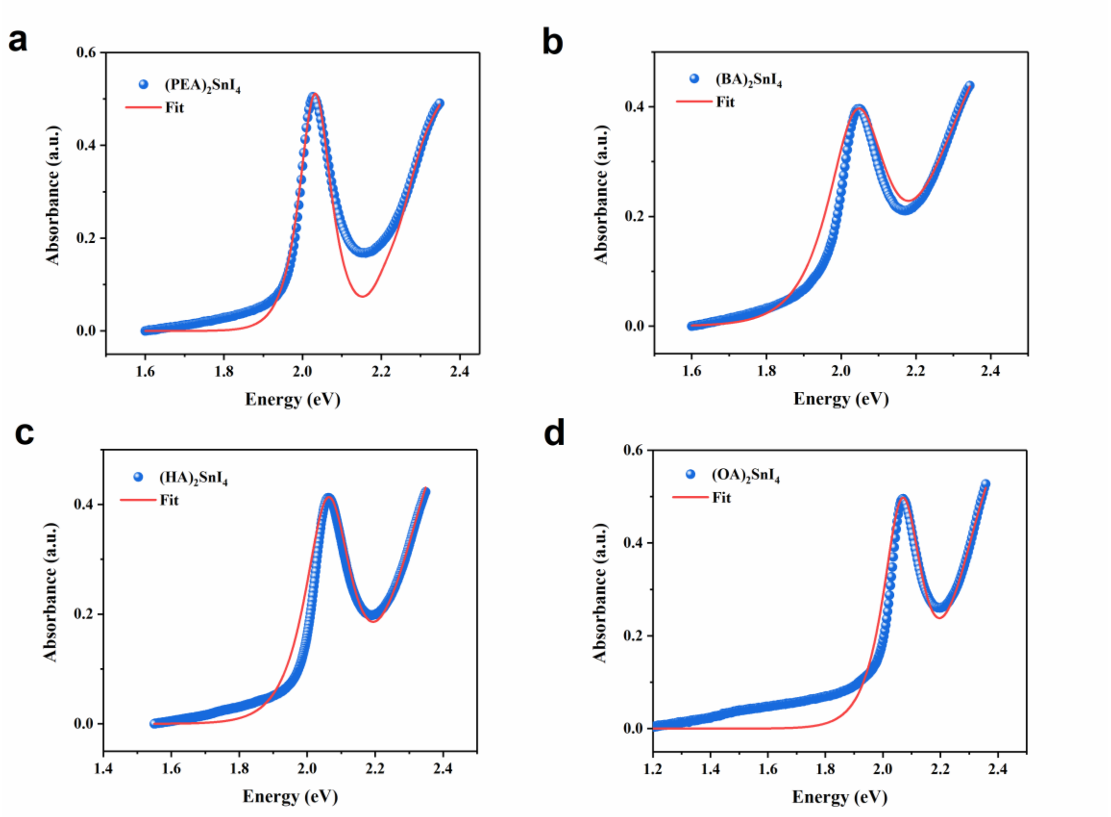

Supplementary Figure 9. Elliott theory of excitonic absorption of the four perovskites: (a) $(PEA)_2SnI_4$, (b) $(BA)_2SnI_4$, (c) $(HA)_2SnI_4$, and (d) $(OA)_2SnI_4$). yielding Exciton binding energy E_b = 213±2 meV, line broadening with Γ = 36±1.5meV, band Gap Eg = 2:275 ±0.001eV for $(PEA)_2SnI_4$. Exciton binding energy E_b = 245±1.6 meV, line broadening with Γ = 70 ±1.5 meV, band Gap Eg = 2:289±0.002 eV for $(BA)_2SnI_4$. Exciton binding energy E_b = 248±1.5 meV, line broadening with Γ = 60 ±1.5 meV, band Gap Eg = 2:31±0.001 eV for $(HA)_2SnI_4$. Exciton binding energy E_b = 236±2meV, line broadening with Γ = 60 ±1.5 meV, band Gap Eg = 2:304±0.001 eV for $(OA)_2SnI_4$.

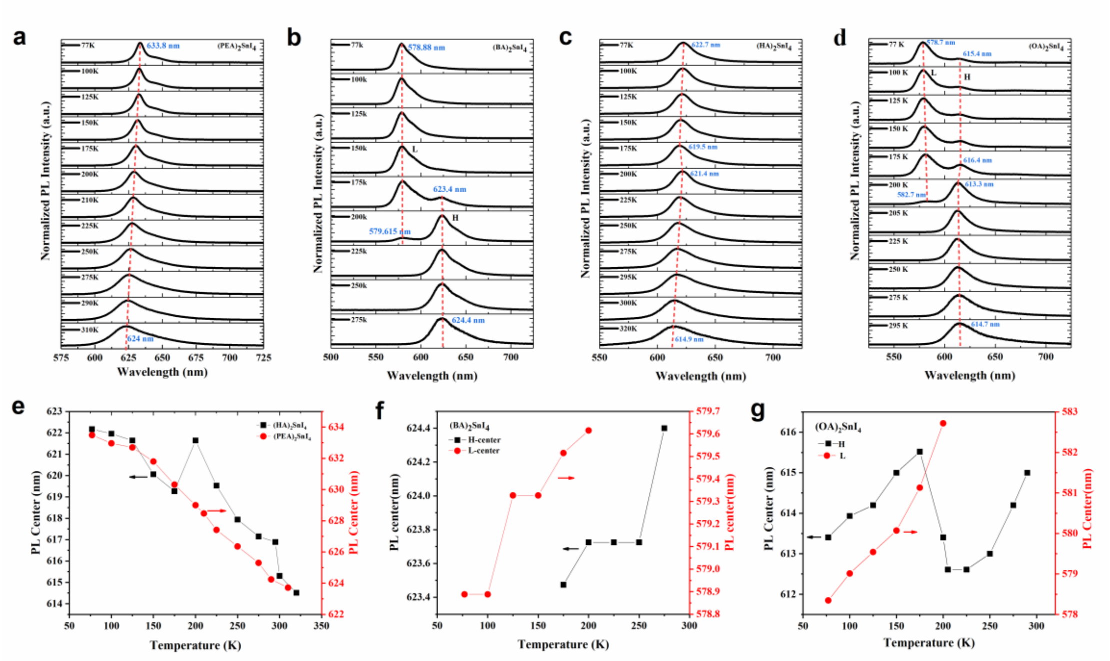

Supplementary Figure 10. Temperature-dependent PL of different perovskite films. The normalized PL spectra of (a) $(PEA)_2SnI_4$, (b) $(BA)_2SnI_4$, (c) $(HA)_2SnI_4$, and (d) $(OA)_2SnI_4$ at different temperatures. Variations of the PL peak positions of (e) $(PEA)_2SnI_4$ and $(HA)_2SnI_4$, (f) $(BA)_2SnI_4$, and (g) $(OA)_2SnI_4$ with temperature (extracted from (a-d)).

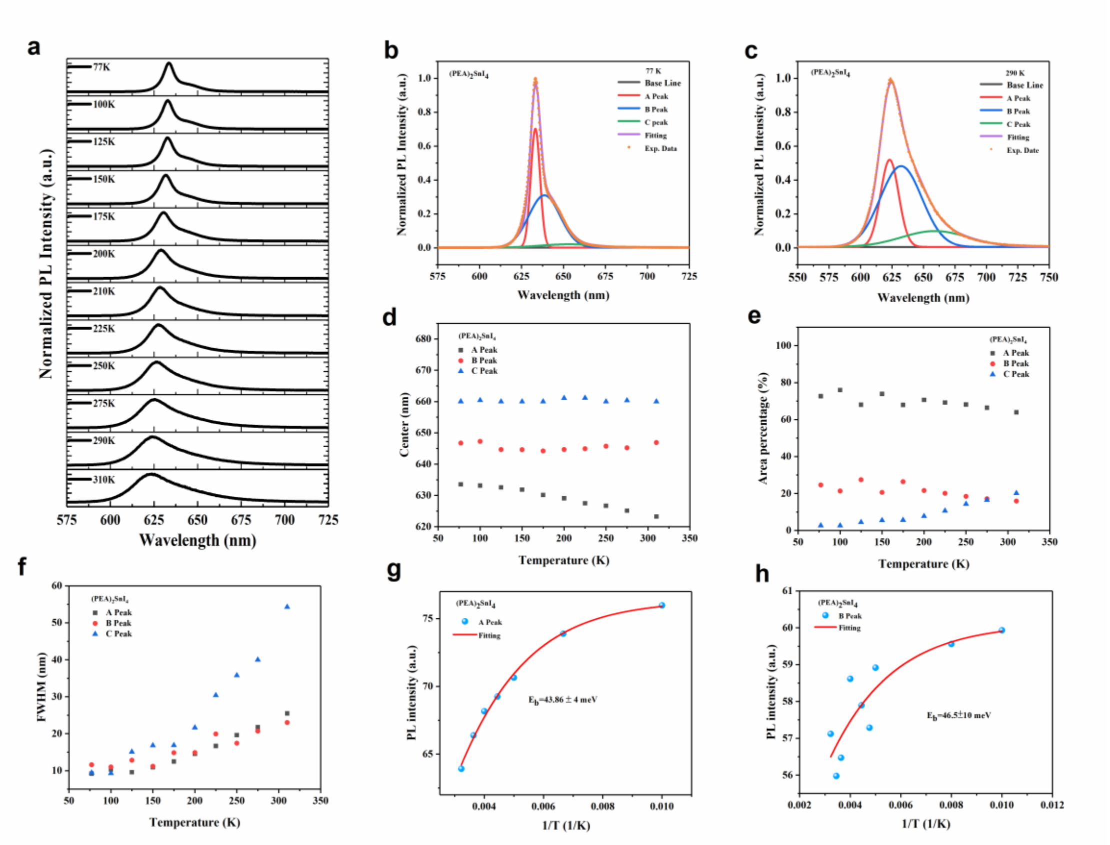

Supplementary Figure 11. The analysis of the influence of temperature on the PL of the $(PEA)_2SnI_4$ samples. (a) Normalized temperature-dependent general PL characteristics. With the decrease in temperature, the PL peak redshifts and the FWHM decreases. Three Gaussian linear PL sub-peaks (*A*, *B*, and *C*) are applied to fit the PL peaks at (b) 77 K and (c) 290 K, where *A* is the free exciton PL peak, *B* is the bound exciton PL peak, and *C* is caused by the photogenerated carriers trapped by the defect state with low absorption cross-sections. (d–f) Change in the center, area ratio, and FWHM of the *A*, *B*, and *C* sub-peaks with temperature. Exciton binding energies of the (g) *A* peak and (h) B peak fitted by the Arrhenius relation.

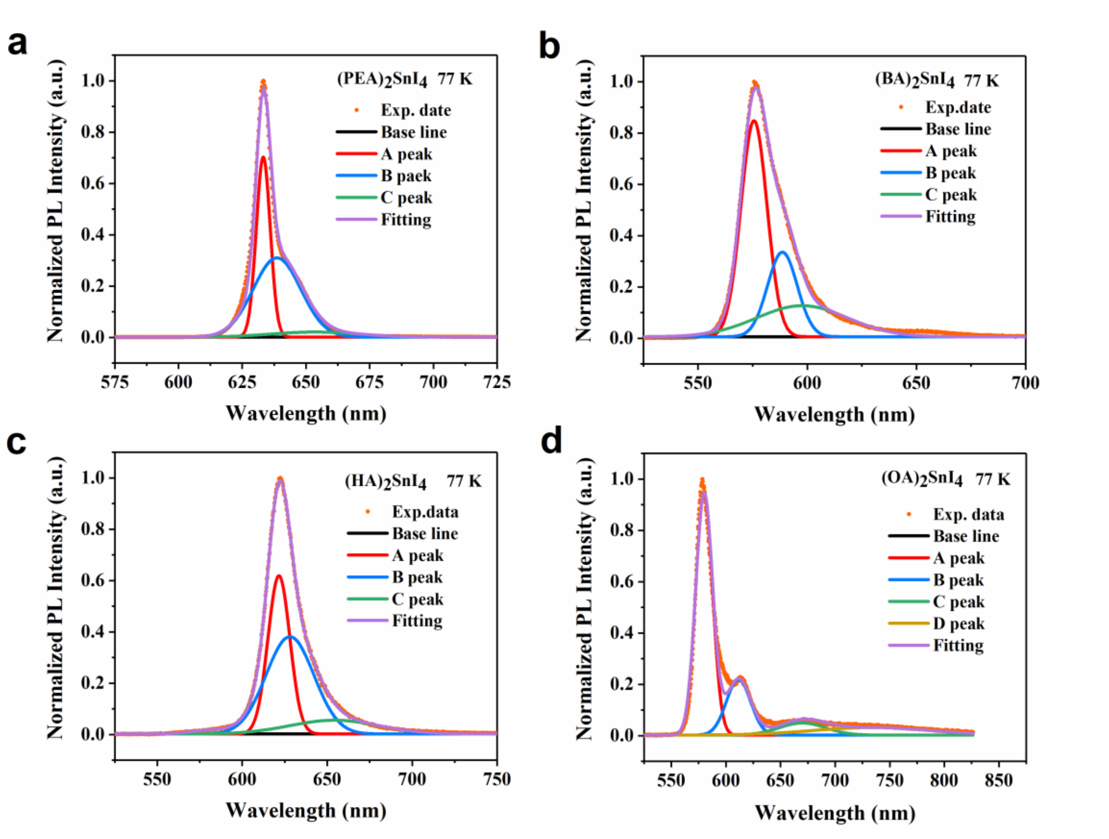

Supplementary Figure 12. PL spectra and their Gaussian fit of four perovskites at 77 K. (a) $(PEA)_2SnI_4$, (b) $(BA)_2SnI_4$, (c) $(HA)_2SnI_4$, and (d) $(OA)_2SnI_4$. Exp. date stands for experimental data. For $(OA)_2SnI_4$ perovskite, four Gaussian peaks are used for fitting.

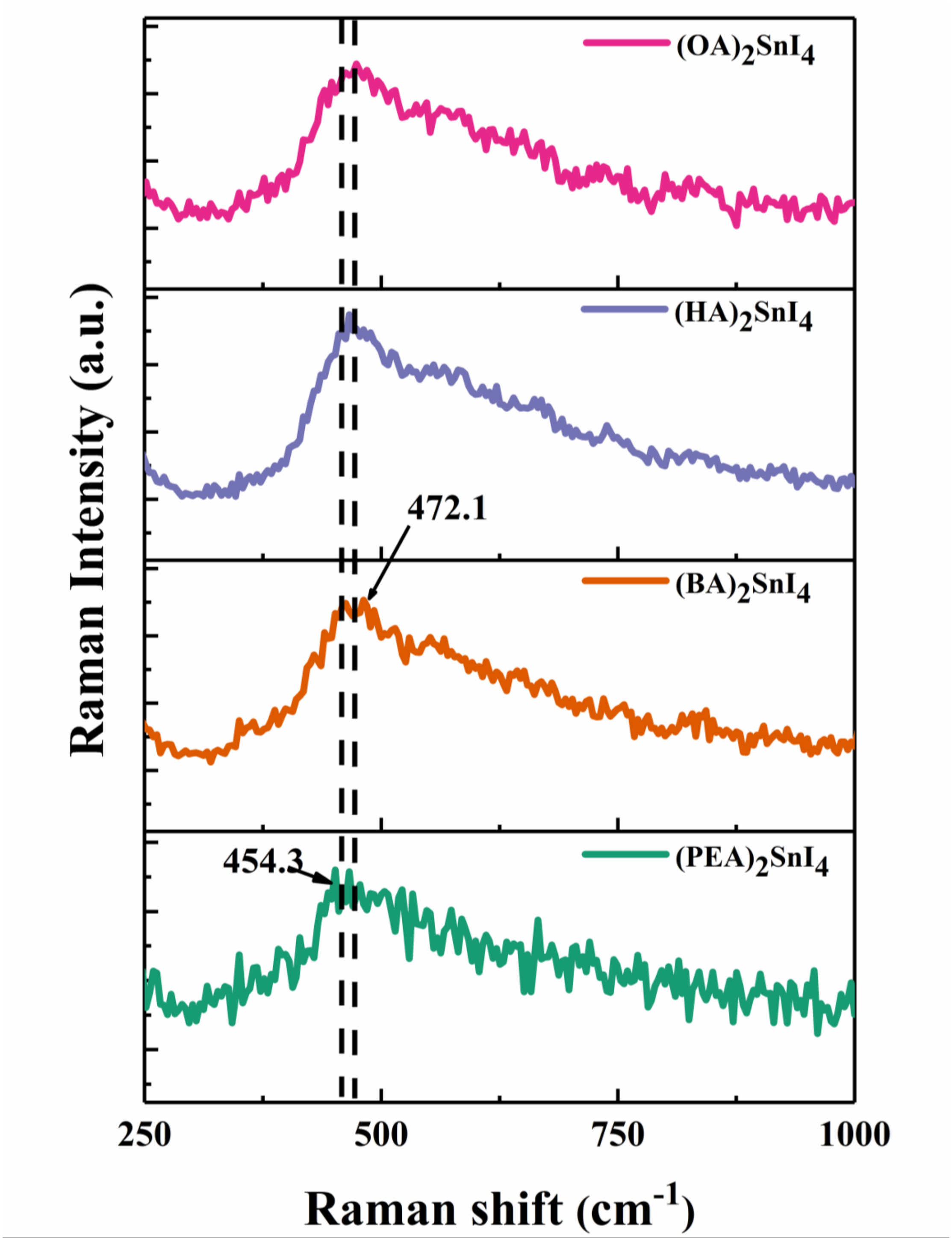

Supplementary Figure 13. Steady-state Raman spectra of the four perovskites (($(PEA)_2SnI_4$, $(BA)_2SnI_4$, $(HA)_2SnI_4$, and $(OA)_2SnI_4$).

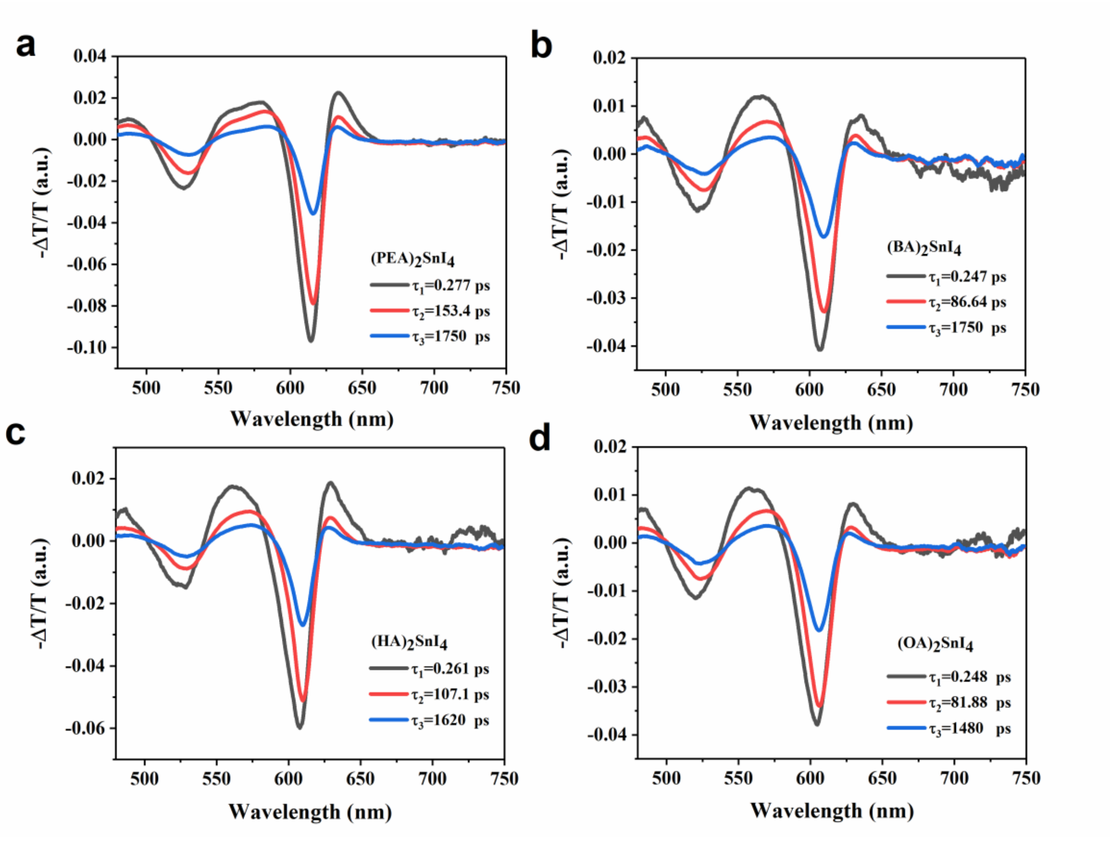

Supplementary Figure 14. Evolution-associated spectra obtained upon global analysis of the TA data of the four perovskites. (a) $(PEA)_2SnI_4$, (b) $(BA)_2SnI_4$, (c) $(HA)_2SnI_4$, and (d) $(OA)_2SnI_4$ thin films with a linear low excitation density (12 μJ cm^{-2}) to avoid high-order recombination processes (exciton–exciton annihilation).

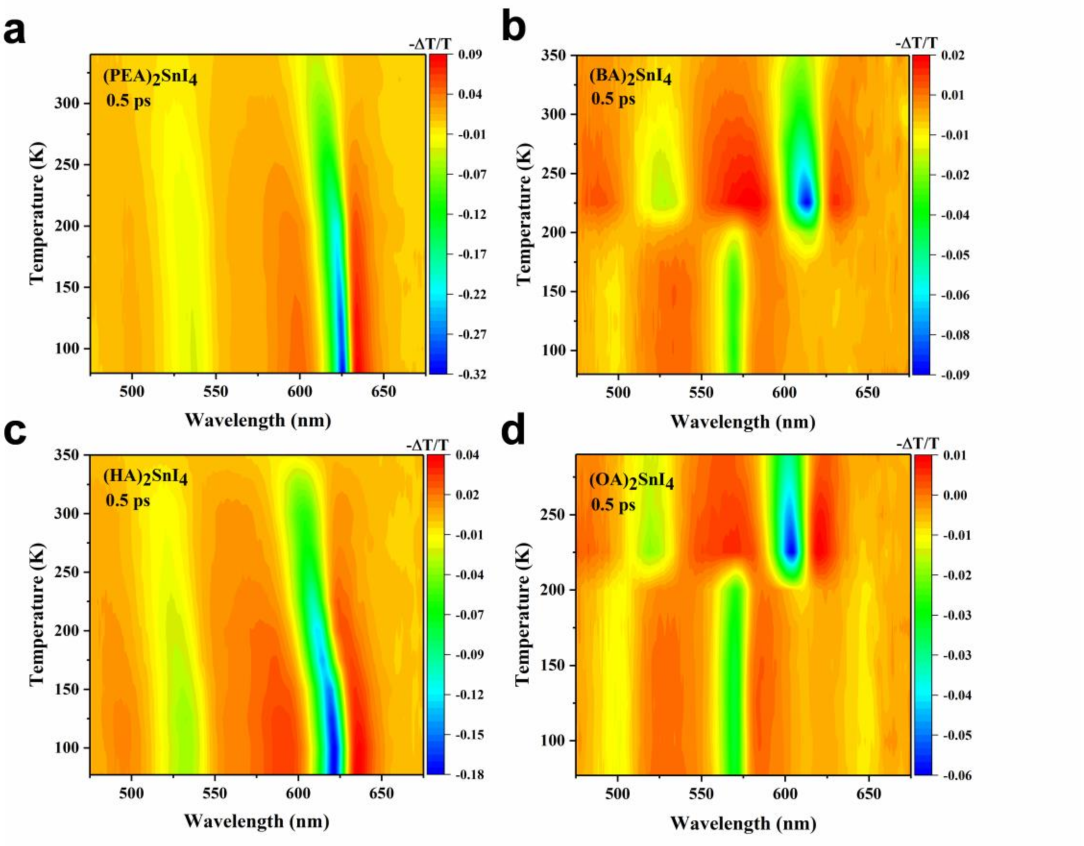

Supplementary Figure 15. Contour map of the temperature dependence of the TA spectra of the four samples with the delay time of 0.5 ps. For $(BA)_2SnI_4$ and $(OA)_2SnI_4$ samples, the TA spectrum changes significantly at temperatures below 200 K, i.e., the bleaching peak is blue-shifted and the intensity of bleaching at the band edge decreases, mainly due to the phase change mentioned earlier. For the $(PEA)_2SnI_4$ and $(HA)_2SnI_4$ samples, the TA spectra were characterized by a redshift with decreasing temperature between 340 K and 77 K and a gradual increase in the intensity of the bleaching peak at the band edge, with no sudden change in the blue shift of the bleaching peak.

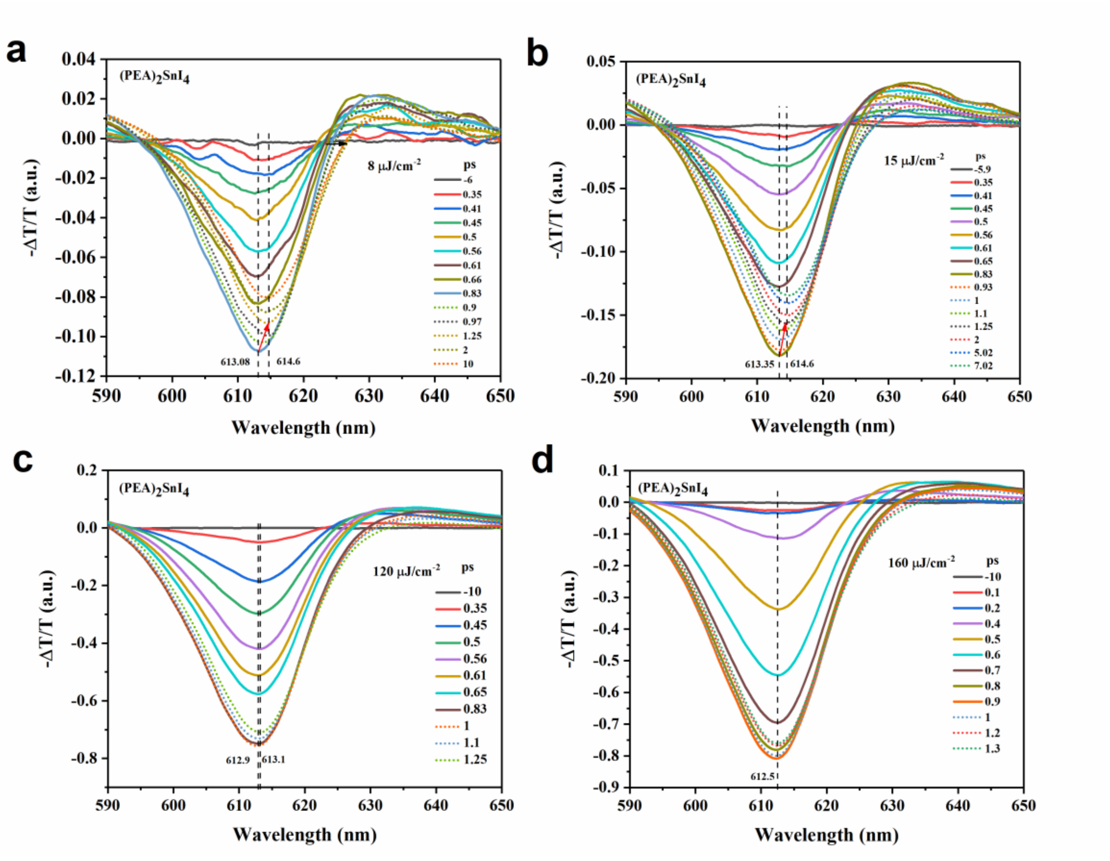

Supplementary Figure 16. Transient dynamic characteristics of the $(PEA)_2SnI_4$ perovskite. The relaxation process of the band edge bleaching peak before 2 ps under the pump fluence of (a) 4 μJ cm^{-2}, (b) 15 μJ cm^{-2}, (c) 120 μJ cm^{-2}, and (d) 160 μJ cm^{-2}. The solid line represents the bleaching peak growth process, and the dotted line represents the band edge bleaching peak relaxation process. The red arrow indicates the direction of movement of the bleach peak.

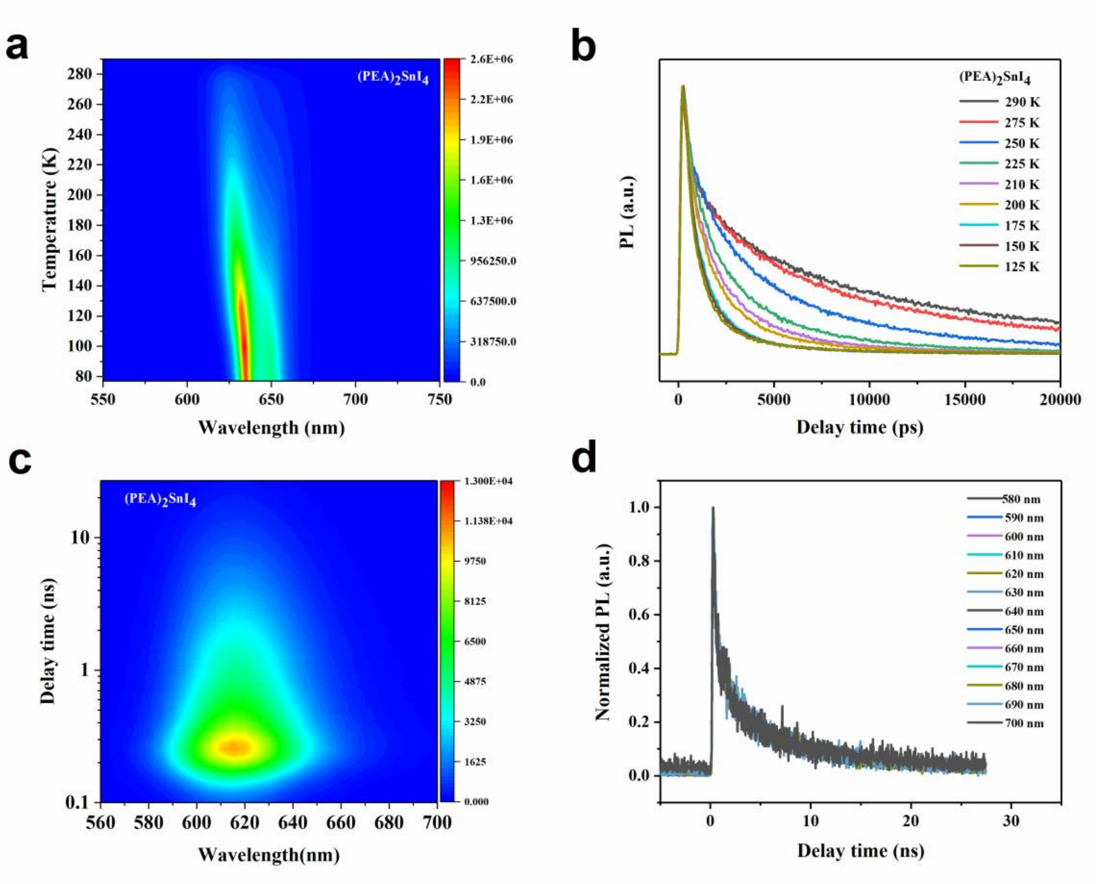

Supplementary Figure 17. Characteristics of time-resolved PL of $(PEA)_2SnI_4$. (a) Temperature-dependent general PL characteristics . (b) Normalized temperature-dependent PL relaxation kinetics under the pump fluence of 1 μJ cm^{-2}. (c) Wavelength-dependent PL relaxation kinetics. (d) Normalized wavelength-dependent PL relaxation kinetics.

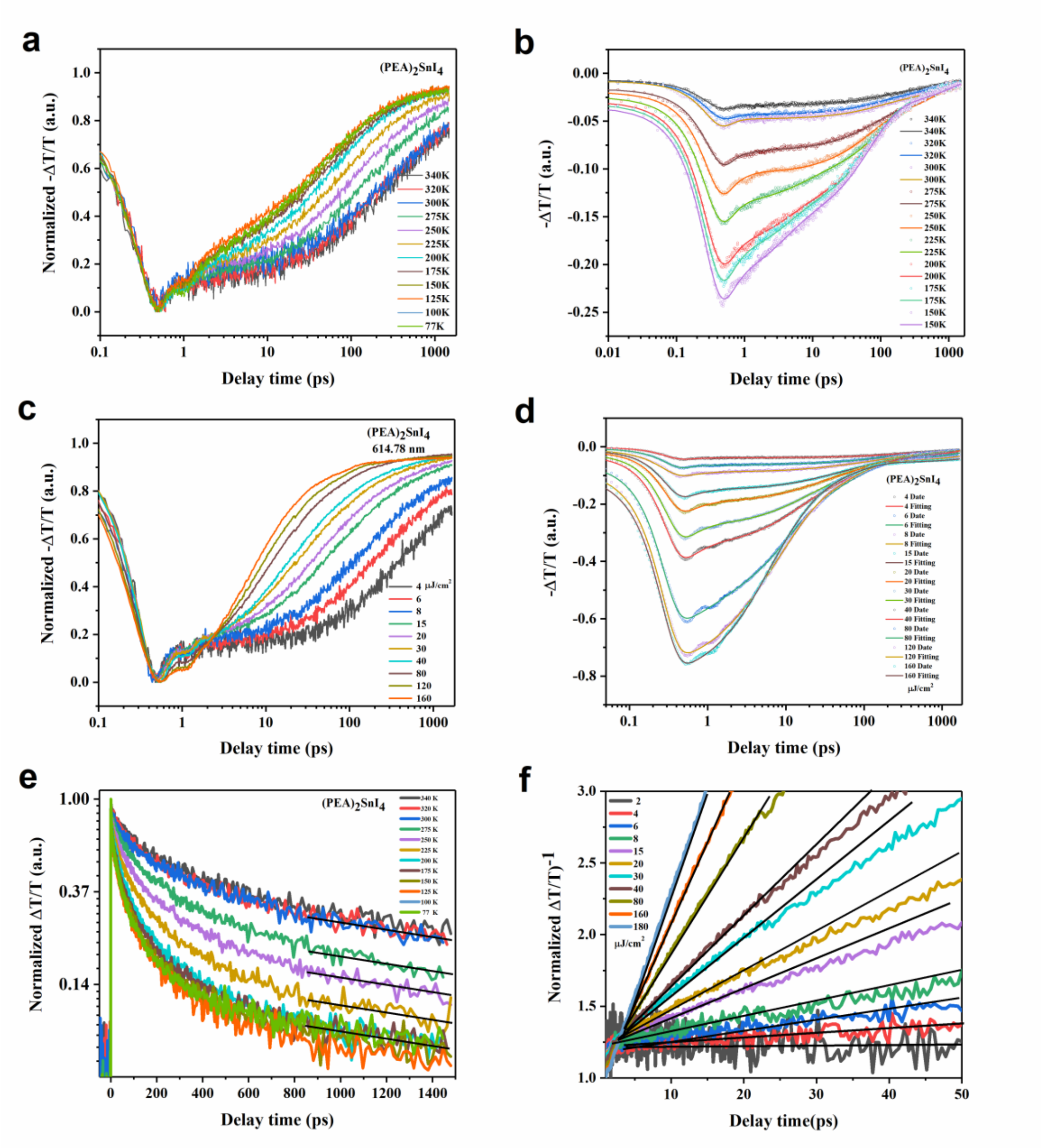

Supplementary Figure 18. The GSB relaxation kinetics of the $(PEA)_2SnI_4$ sample. (a) Temperature-dependent normalized GSB relaxation kinetics under the pump fluence of 4 μJ cm^{-2} and (c) pump fluence-dependent normalized GSB relaxation kinetics (614.78 nm) at room temperature. The fitting results of (b) temperature-dependent and (d) pump fluence-dependent GSB relaxation kinetics (614.78 nm). (e) Temperature-dependent normalized GSB relaxation kinetics under the pump fluence of 4 μJ cm^{-2} plotted on a semilog scale. (f) The inverse of pump fluence-dependent normalized GSB relaxation kinetics (614.78 nm) at room temperature. The black solid lines in (e) and (f) are linear fittings, representing the single-particle interaction process and the two-body interaction process in the relaxation process, respectively.

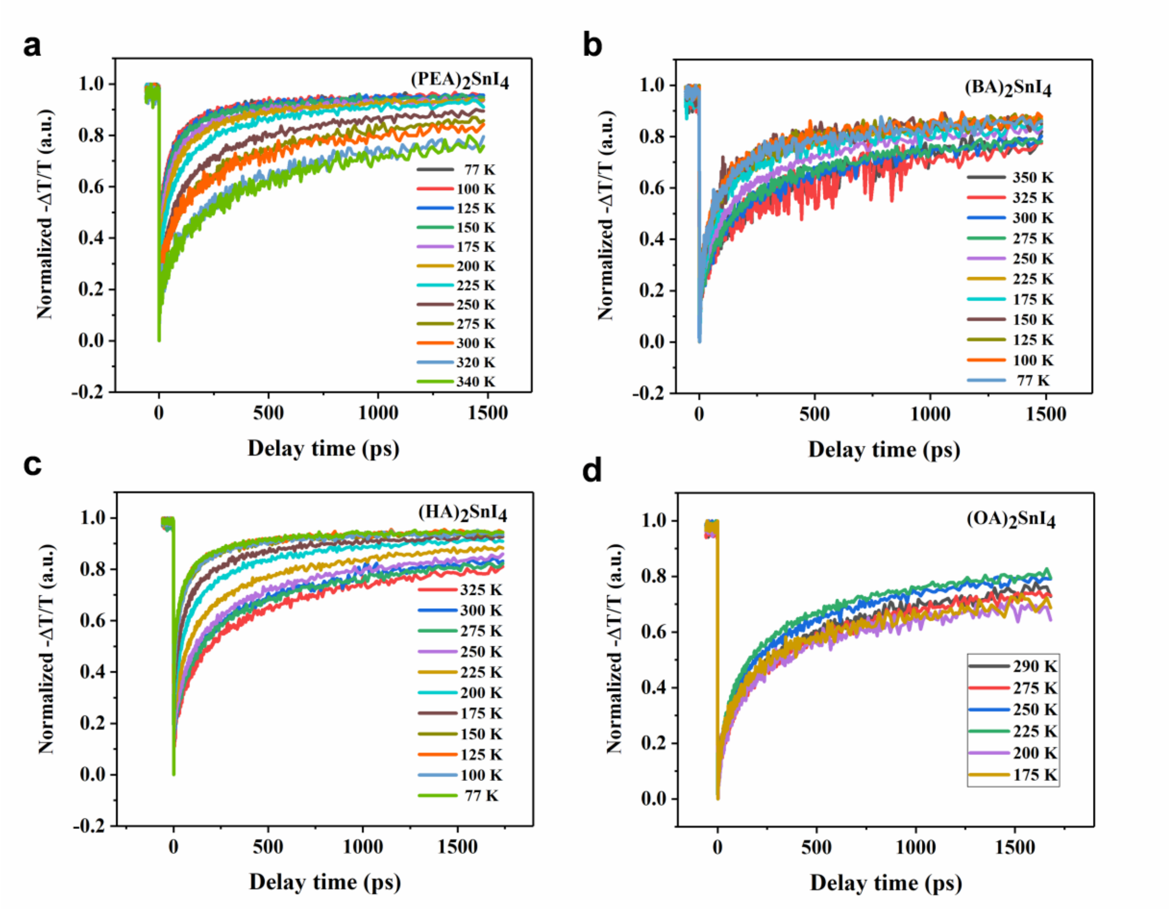

Supplementary Figure 19. Temperature-dependent normalized GSB relaxation kinetics of the four samples.

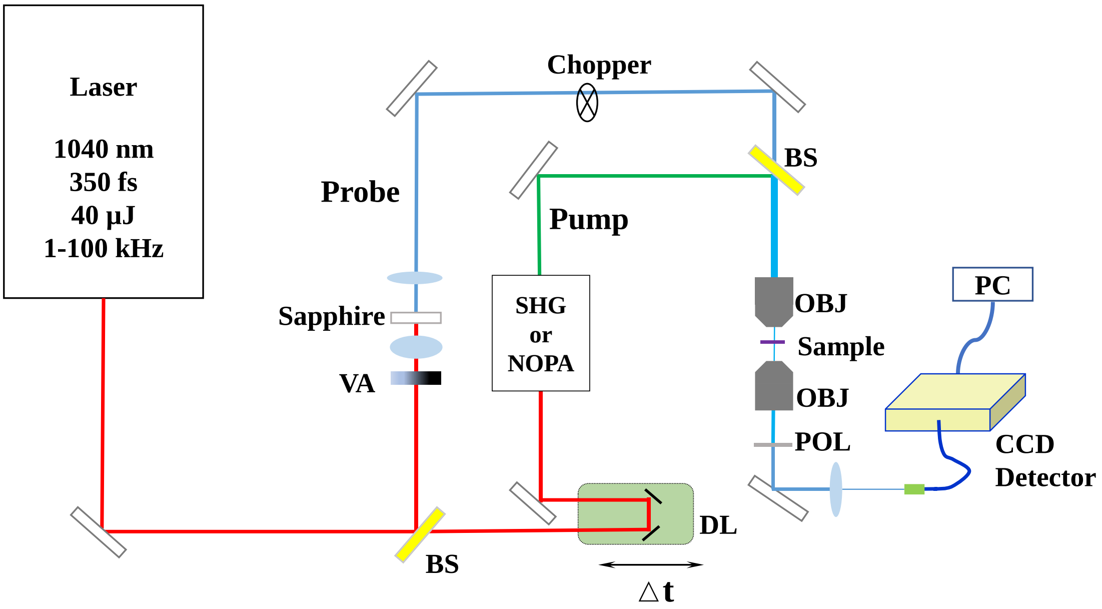

Supplementary Figure 20. Schematic diagram of the TA system. VA: adjustable attenuator, BS: beam splitter, DL: delay stage, OBJ: Optical microscopic objective lens, and POL: optical polarizer.

Supplementary Table 1. SCXRD data of $(PEA)_2SnI_4$, $(BA)_2SnI_4$, $(HA)_2SnI_4$, and $(OA)_2SnI_4$ single crystals at 275 K and 125 K.

**Empirical formula**	**$(PEA)_2SnI_4$**		**$(BA)_2SnI_4$**		**$(HA)_2SnI_4$**		**$(OA)_2SnI_4$**	
**Temperature [K]**	275	125	275	125	275	125	275	125
**Formula weight**	1741.32	870.66	774.58	774.58	830.68	830.68	886.79	886.79
**Crystal system**	Triclinic	Triclinic	Orthorhombic	Orthorhombic	Orthorhombic	Monoclinic	Orthorhombic	Monoclinic
**Space group**	P-1	P-1	Pbca	Pbca	Pbca	$P2_1/c$	$P2_12_12_1$	$P2_1/c$
***a* [Å]**	8.6785(12)	8.6344(7)	8.8378(13)	8.475(3)	8.8685(4)	16.1466(13)	8.6303(11)	18.729(8)
***b* [Å]**	8.6808(12)	8.6468(8)	8.6456(13)	8.895(3)	8.6172(3)	8.8416(8)	37.562(5)	8.877(4)
***c* [Å]**	32.800(5)	32.264(3)	27.587(4)	26.135(9)	32.7079(15)	8.6125(6)	8.9275(11)	8.397(4)
**α [°]**	84.715(4)	85.261(3)	90	90	90	90	90	90
**β [°]**	84.742(4)	85.123(3)	90	90	90	92.126(7)	90	96.707(10)
**γ [°]**	89.618(4)	89.512(3)	90	90	90	90	90	90
**Volume/$Å^3$**	2450.2(6)	2391.9(4)	2107.9(5)	1970.1(12)	2499.59(18)	1228.69(17)	2894.0(6)	1386.5(10)
**Z**	2	4	4	4	4	2	4	2
**ρ_{calc} (g/cm^3)**	2.360	2.418	2.441	2.611	2.207	2.245	2.035	2.124
**μ/mm^{-1}**	6.084	6.233	38.144	7.548	5.957	6.060	27.847	5.377
**Independent**	8638	10083	1858	3092	2748	2181	6394	3105

**reflections**	[R_{int} = 0.0806]	[R_{int} = 0.1248]	[R_{int} = 0.0599]	[R_{int} = 0.1254]	[R_{int} = 0.0545]	[R_{int} = 0.1429]	[R_{int} = 0.0599]	[R_{int} = 0.0713]
**Goodness-of-fit on F^2**	1.093	1.096	1.023	1.107	1.113	1.173	1.006	1.042
**Final R indexes [all data]**	R_1 = 0.1979, wR_2 = 0.2619	R_1 = 0.1031, wR_2 = 0.2037	R_1 = 0.0587, wR_2 = 0.1594	R_1 = 0.0897, wR_2 = 0.2377	R_1 = 0.0642, wR_2 = 0.0937	R_1 = 0.1047, wR_2 = 0.2294	R_1 = 0.1405, wR_2 = 0.3140	R_1 = 0.1256, wR_2 = 0.2990
**Average Sn-I [Å]**	3.1398	3.1272	3.1528	3.1629	3.1529	3.1644	3.1653	3.1398

Supplementary Table 2. Absorption band edge, PL center peak position, Stokes shift (Δ_s), and PL FWHM of the four perovskite polycrystalline film samples. The absorption band edge is obtained by the second-derivative analysis of the linear absorption spectra.

Sample	E_g (eV)	PL Center (eV)	Stokes Shift Δ_s (meV)	FWHM (meV)
$(PEA)_2SnI_4$	2.02	1.987	35	91
$(BA)_2SnI_4$	2.04	1.997	42	125
$(HA)_2SnI_4$	2.05	2.01	43	100
$(OA)_2SnI_4$	2.06	2.016	43	120

Supplementary Table 3. Linewidth parameters for perovskites reported by other experimental studies.

Sample	Γ_0 (meV)	Γ_{LO} (meV)	E_{LO} (meV)	γ_{ac}(meV/K)
$FAPbI_3$[1]	19±1	40±5	11.5±1.2	
$FAPbBr_3$[1]	20±1	61±7	15±1.4	19±1
$FAPbBr_3$[2]	18.1	34.8	18	0.078
$MAPbI_3$[1]	26±2	40±2	11.5	
$MAPbI_{3-x}Cl_x$[3]	37	22	10	
$MAPbBr_3$[1]	32±2	58±2	15.3	19±1
$FAPbBr_3$[2]	18.1	34.8	18	
$MASnI_3$[4]	45.2±1.1	53.8±7.6	21.6±2.4	
BA_2PbI_4[5]	13.2±0.4	34.2±0.3	10.9	
HA_2PbI_4[5]	18.7±0.3	55.8±0.4	17	

Supplementary Note 1. Analysis of SCXRD data of 2D perovskites

Due to the symmetry and size effects of different organic cations, the as-synthesized 2D perovskites were crystallized in different space groups. At 275 K, $(PEA)_2SnI_4$ was crystallized in a triclinic space group of P-1, with lattice parameters of a = 8.6785(12) Å, b = 8.6808(12) Å, c = 32.800(5) Å, α = 84.715(4), β = 84.742(4) and γ = 89.618(4). While for the BA-, HA- and OA-perovskites with saturated alkyl-ammonium cations hosted among the perovskite layers, orthorhombic perovskites with similar well-aligned octahedral building units were observed. $(BA)_2SnI_4$ was crystallized in an orthorhombic space group of Pbca, with lattice parameters of a = 8.8378(13) Å, b = 8.6456(13) Å, and c = 27.587(4) Å. $(HA)_2SnI_4$ was crystallized in an orthorhombic space group of Pbca, with lattice parameters of a = 8.8685(4) Å, b = 8.6172(3) Å and c = 32.7079(15) Å. $(OA)_2SnI_4$ was crystallized in an orthorhombic space group of $P2_12_12_1$, with lattice parameters of a = 8.6303(11) Å, b = 37.562(5) Å and c = 8.9275(11) Å. Here, it was observed that the longer alkyl-ammonium chain led to the extended spacing between the 2D inorganic layers. At 125 K, due to the contraction effects in the cooling process, the unit cell of four different types of 2D Sn-based perovskites shrunk, leading to the decreasing of all the a, b, and c axis. The unit cell of $(PEA)_2SnI_4$ without the existence of phase transition in the measured temperature range was evolved from a space group of P-1, with lattice parameters of a = 8.6785(12) Å, b = 8.6808(12) Å, c = 32.800(5) Å, α = 84.715(4), β = 84.742(4) and γ = 89.618(4) to space group of P-1, with lattice parameters of a = 8.6344(7) Å, b = 8.6468(8) Å, c = 32.264(3) Å, α = 85.261(3), β = 85.123(3) and γ = 89.512(3). However, for the alkyl-ammonium chain structures, obvious phase transitions were observed. The unit cell of $(BA)_2SnI_4$ was evolved from a space group of Pbca, with lattice parameters of a = 8.8378(13) Å, b = 8.6456(13) Å and c = 27.587(4) Å to space group of Pbca, with lattice parameters of a = 8.475(3) Å, b = 8.895(3) Å, and c = 26.135(9) Å. The orthorhombic–orthorhombic phase transition observed here is a first-order solid–solid phase transition[5, 6]. While for $(HA)_2SnI_4$ and $(OA)_2SnI_4$, orthorhombic–monoclinic

phase transitions were involved. The unit cell of $(HA)_2SnI_4$ was evolved from a space group of Pbca, with lattice parameters of a = 8.8685(4) Å, b = 8.6172(3) Å and c = 32.7079(15) Å to space group of $P2_1/c$, with lattice parameters of a = 16.1466(13) Å, b = 8.8416(8) Å, c = 8.6125(6) Å, and β = 92.126(7). The unit cell of $(OA)_2SnI_4$ was evolved from a space group of $P2_12_12_1$, with lattice parameters of a = 8.6303(11) Å, b = 37.562(5) Å and c = 8.9275(11) Å to space group of $P2_1/c$, with lattice parameters of a = 18.729(8) Å, b = 8.877(4) Å, c = 8.397(4) Å, and β = 96.707(10).

Supplementary Note 2. UPS analysis of the different perovskites

Here the valence band (VB) levels are calculated using the formula below. The Fermi level (E_F) energies are obtained from the cutoff region: 3.27 eV (BA), 3.19 eV (HA), 3.28 eV (OA), and 2.95 eV (PEA); the bandgaps (E_g) are determined from the $(Ahv)^2 \sim hv$ curves (Supplementary Fig. 6b): 1.973 eV (BA), 1.998 eV (HA), 2.004 eV (OA), and 1.969 eV (PEA).

$$E_F - E_v = E - hv \; (hv \; = \; 21.22 \; eV), \qquad (1)$$

Where E is the value marked at the edge of the VB edge region, and the energy differences between the VBM and E_F are 1.0, 0.66, 0.77, and 0.53 eV for $(PEA)_2SnI_4$, $(BA)_2SnI_4$, $(HA)_2SnI_4$, and $(OA)_2SnI_4$ samples, respectively.

Supplementary Note 3. Analysis of the transition process of the PB2 bleaching peak at 520nm

We attributed the absorption peak at ~520 nm to the intraband transition of SnI_4 inorganic layers by combining reported research results with the results of pump energy-dependent transient absorption (TA) spectroscopy. It is mainly because the first excitation energy of PEA^+, BA^+, HA^+, and OA^+ cations lie in the UV energy range[7], which is much higher than the lowest optical excitation peak energy of the perovskite layer. The excitons in the inorganic perovskite lattice can not transfer to spin-triplet Frenkel excitons states located on the organic cation[7]. Since A_2SnI_4 perovskite (A = PEA^+, BA^+, HA^+, and OA^+) have similar linear absorption and TA spectra characteristics, we selected $(PEA)_2SnI_4$ as a representative for an explanation. The details are shown below:

In 2D layered halide organic perovskites (LHOPs), the perovskite layer is the dominant component of band-edge absorption. Therefore, a way to demonstrate energy transfer would be the observation of triplet emission from the organic spacer layer[8, 9]. Some LHOPs have demonstrated the ability to induce energy transfer from perovskite layer exciton states to low energy spin-triplet exciton states in the organic layer[9, 10, 11]. When the lowest excitation energy (E) of the exciton in the perovskite layer aligns with the first triplet (T_1) excitation energy of the exciton in the organic layer, charge transfer from the perovskite to the organic layer may occur. After transfer, the T_1 excitation energy in the organic layer relaxes to a lower T_1^* energy due to enhanced short-range atomic deformation, thus reaching optimal triplet molecular geometry[7]. Through theoretical calculations, Neukirch et al. systematically studied organic spacer and perovskite layer pairings for possible transfer of the Wannier excitons from the inorganic perovskite lattice to spin-triplet Frenkel excitons located on the organic cations and successfully identify ten organic spacer candidates for possible pairing with perovskite layers of specific halide composition to achieve triplet light emission across the visible energy range. From their calculations, it is clear that the T_1 energy of PEA^+

remains in a narrow range between 4.43 and 4.46 eV, which is greater than the lowest optical excitation peak energy (2.5 eV) of perovskite layer in the $(PEA)_2PbI_4$. The absorption spectrum shows that the lowest optical excitation peak energy of SnI_4 in $(PEA)_2SnI_4$ is smaller than that of PbI_4 in $(PEA)_2PbI_4$ (2.02 eV *vs* 2.5 eV), so we can conclude that the exciton in SnI_4 cannot be transferred to organic cation PEA^+. For the alkylammonium chains, it can not be excited by a photon in the visible region[12], so we can also conclude that the exciton in SnI_4 cannot be transferred to organic cation the alkylammonium chains.

In the pump energy-dependent TA spectroscopy experiments, where we used the wavelength of a pump at 400 nm, the wavelength of the pump at 613 nm with resonant band-edge absorption, and the wavelength of the pump at 630 nm below the bandgap, we found the existence of photobleaching peaks at ~523 nm (PB1) and ~613 nm (PB2) in the TA spectra of these three different pump energies (Supplementary Fig. 4.). The relaxation kinetics of A_2SnI_4 (A = PEA^+, BA^+, HA^+, and OA^+) obtained by low pump fluence have been fitted globally with three components (Supplementary Fig. 14). We find that PB1 has the same relaxation characteristics and lifetime as PB2, and hence it is not consistent with the occurrence of the CT transition. In Supplementary Fig. 5, the two PB peaks are generated almost simultaneously when excited at 400 nm. However, the PB1 peak first reaches the maximum and then decreases, and the PB2 peak reaches a maximum with a delay of about 0.1 ps compared to the PB1 peak, which is more consistent with the intraband transitions[13].

Combined with the above analysis, the PB1 peak is not the charge transfer transition between the organic spacer and the inorganic layers, but an intraband transitions process in the perovskite layer in the $(PEA)_2SnI_4$.

Supplementary Note 4. Fitting of absorption spectra based on Elliott's formula

For the estimation of the binding energy (E_b) of Wannier exciton contained in the direct bandgap semiconductor, an effective method is to fit the band-edge absorption spectrum using the Elliott formula[14]. According to Elliott theory of two-dimensional system[15, 16, 17], the absorption coefficient can be expressed as

$$\begin{aligned}\alpha(\omega) &= \alpha_{\text{exc}} + \alpha_{\text{cont}} \\ &= \alpha_0 \left[\sum_{n=1}^{\infty} \frac{4E_0}{\left(n-\frac{1}{2}\right)^3} \operatorname{sech}\left(\frac{\hbar\omega - E_g + \frac{E_0}{(n-1/2)^2}}{\Gamma_{ex}} \right) \right. \\ &+ \int_{E_g}^{\infty} \operatorname{sech}\left(\frac{\hbar\omega - x}{\Gamma_c}\right) \frac{2}{1+\exp\left(-2\pi\sqrt{\frac{E_0}{\hbar\omega - E_g}}\right)} \\ &\left. \times \frac{1}{1-(8\alpha m^*/\hbar^4)(x-E_g)} dx \right] \end{aligned} \tag{2}$$

Here, the first term on the right side of the equation is the excitonic absorption component below the band edge, and the second term is the free-carrier continuum absorption component. A term to correct for the non-parabolic band dispersion is introduced. E_g is the single-particle bandgap, m^* is the exciton reduced mass, a hyperbolic secant function to account for a phenomenological broadening Γ_{ex} and Γ_c represent exciton and free carrier transitions, respectively.

Supplementary Note 5. The analysis of temperature-dependent PL

The normalized PL spectra of the four materials at different temperatures are provided in Supplementary Fig. 10 The PL spectra of the four perovskite materials present asymmetric characteristics at room temperature with the low-energy PL tails in a wide energy range. As the temperature decreases, the FWHM of the PL decreases, and the symmetry increases, indicating that the PL of the samples is induced by multiple luminescent components. Recent studies have shown that there exist spectrally distinguishable double PL peaks in the 2D perovskite single crystal. Hence, three Gaussian peaks are used to fit the overall PL spectra: Gaussian peaks **A** and **B** are attributed to the excitons, whereas the lowest energy peak **C** is attributed to the shallow trap state[18] (Supplementary Fig. 11 and Supplementary Fig. 12). In particular, the trailing spectral width of PL is more and more serious in $(HA)_2SnI_4$, $(BA)_2SnI_4$, and $(OA)_2SnI_4$, especially in $(BA)_2SnI_4$, and $(OA)_2SnI_4$ at 77 K when the phase transition causes PL blue shift with existing PL trailing at 640-700 nm, and $(OA)_2SnI_4$ is more obvious (Supplementary Fig.12).

As the temperature decreases, except for the blueshift (2 nm) of the PL peak of $(HA)_2SnI_4$ at 200 K, the PL peaks of $(PEA)_2SnI_4$ and $(HA)_2SnI_4$ exhibit an almost monotonous redshift. From the viewpoint of thermal expansion, the bandgap of traditional semiconductor materials increases as the temperature decreases, including MoS_2[19] and GaAs[20]. This is because, in most semiconductors, the bandgap exists between the *p* orbitals of anions and the *s* orbitals of cations (Supplementary Fig. 7.). When the lattice constant decreases, the atom is close, the covalent interaction between the two orbitals increases, and the bandgap increases. However, for the A_2SnI_4 structure material, the VBM mainly comprised the 5*s* orbital of Sn and the 5*p* orbital of I, and the conduction band minimum mainly comprised the empty 5*p* orbital of Sn. A decrease in the lattice constant strengthens the interaction between the 5*s* orbitals of Sn and the 5*p* orbitals of I, which results in an increase in the VB width and an increase in the VB energy. This explains the abnormal bandgap narrowing behavior. However, the

characteristics of the temperature-dependent PL of the $(BA)_2SnI_4$ and $(OA)_2SnI_4$ samples are different (Fig. 3 and Supplementary Fig. 10,). With the decrease in temperature, the original PL peak (denoted as peak H) exhibits a slight blueshift of approximately 0.7 nm for $(BA)_2SnI_4$ and 1.4 nm for $(OA)_2SnI_4$. A new PL peak (denoted as peak L) appears at the high-energy edge of the PL peak (H) at about 200 K, and with the further decrease in temperature, the L PL peak blueshifts by 0.4 nm for $(BA)_2SnI_4$ and 4 nm for $(OA)_2SnI_4$. Meanwhile, the L PL peak intensity exceeds the H PL peak intensity, and the H PL peak gradually decreases or until reaching zero, which means that the photogenerated carriers recombine by radiation transfer from the low energy band edge position to the high energy luminous position as the temperature decreases. This phenomenon has also been observed in $MAPbI_3$ and $MAPbBr_3$ perovskites, which undergoes sharp and discontinuous phase transitions from the tetragonal phase to the orthorhombic phase due to the significantly decreased rotational freedom of the organic cation[1, 21]. As a result, the band structure has changed, and the optical bandgap of the material in the orthorhombic phase is larger than that in the tetragonal phase for 3D perovskites[22, 23]. Combined with our SCXRD experimental results at low temperature (Supplementary Table 1), the new L PL peaks of $(BA)_2SnI_4$ and $(OA)_2SnI_4$ at 200 K are attributed to the structural phase transition. Therefore, in the low-temperature phase, the high-energy peak (L peak) is assigned to the band edge luminescence, whereas the H PL peak with low energy belongs to trapped or bound charge carrier pairs originating from the high-temperature phase in $(BA)_2SnI_4$ and $(OA)_2SnI_4$. This view has been confirmed in some studies that demonstrate the coexistence of crystalline phases and attribute it to the strain imposed by the substrate on the perovskite thin films[24].

Supplementary Note 6. exciton-phonon coupling model

For most semiconductors, using the first-order perturbation theory, the temperature-dependent characteristics of the PL peak can be simplified to three scattering mechanisms:

$$\Gamma(T) = \Gamma_0 + \gamma_{ac}T + \frac{\Gamma_{LO}}{(e^{E_{LO}/K_BT}-1)} + \Gamma_{imp}e^{-E_{imp}/k_BT}, \quad (3)$$

where the first term on the right side (Γ_0) represents an inhomogeneous broadening independent of the temperature, which is attributed to the scattering process caused by the crystal disorder[1]. The second and third terms are the temperature-dependent homogeneous broadenings, attributed to the exciton–phonon scattering. In other words, excitons are scattered with acoustic phonons and longitudinal optical (LO) phonons, where γ_{ac} is the coupling strength of the electron and acoustic phonon scattering, Γ_{LO} is the Fröhlich coupling strength of the exciton scattering with LO phonons, and E_{LO} is the energy of the LO phonon. The scattering intensity between the excitons and phonons is generally proportional to the number of phonons. The number of phonons adopts the Bose–Einstein distribution characteristics[25], i.e., as the temperature decreases, the number of phonons decreases, and the scattering effect weakens. Therefore, the FWHM of PL decreases with the temperature. Since the energy of acoustic phonons is considerably smaller than K_BT, the homogeneous broadening caused by the scattering between acoustic phonons and excitons can be approximated as a linear change with temperature[1, 4, 5, 26, 27, 28]. To qualitatively distinguish the contributions between the acoustic and optical phonons, it is necessary to consider the gradient difference of the linewidth curve at low temperatures. According to the model, at a low temperature, the optical phonon causes a gradient of 0, whereas the acoustic phonon causes a gradient greater than 0. By carefully observing that the gradient change in the FWHM of the $(PEA)_2SnI_4$ samples approaches 0 at low temperatures below 77 K (Fig. 3), the scattering between the acoustic phonons and excitons can be ignored, and γ_{ac} is set as 0 in the fitting. This judgment is consistent with the results of polar inorganic semiconductors, i.e., acoustic phonon scattering is relatively negligible compared with

the optical phonon scattering at room temperature[1]. The last term describes the inhomogeneous broadening caused by ionized impurities (coupling Γ_{imp}), where the impurities with the binding energy, E_{imp}, are completely ionized[25, 29, 30]. In the fitting process, this model does not consider the change in the phonon energy with temperature and the use of a single phonon energy fitting; consequently, the fitting results have some limitations. However, it plays a guiding role in the understanding of the scattering between excitons and phonons in perovskite materials.

Supplementary Note 7. Band filling and dynamic Burstein–Moss shift

We observe a pump fluence-dependent blueshift and a broadening of the bandedge bleach peak (Fig. 7 and Supplementary Fig. 16). This broadening is indicative of carrier accumulation and can be interpreted in the light of the dynamic Burstein-Moss shift[31]. As the photogenerated carriers thermalize, they fill the energy positions at the conduction and valence bandedges. Due to the Pauli exclusion principle, this occupation of the bandedge states forces a higher-energy optical transition, causing a blue shift in the bleaching peak. The photogenerated carrier-induced blueshift can be modeled according to the equation:[32]

$$\Delta E_{\mathrm{g}}^{\mathrm{BM}} = \frac{\hbar^2}{2\boldsymbol{m}^*}(3\pi n)^{2/3} \propto F^{2/3} \tag{4}$$

Where $\Delta E_{\mathrm{g}}^{\mathrm{BM}}$ is the increase in the optical bandgap due to the Burstein–Moss band filling effect, m^* is the reduced effective mass, $\hbar$ is the reduced Planck constant. n is the density of the photoinduced exciton that is proportional to the pump fluence F.

Supplementary Note 8. Pump fluence dependence of TA lifetime

The pump fluence dependence of the lifetime (τ_4) can be well explained by the two-body recombination[31, 33, 34]:

$$\frac{dn}{dt} = kn^2 \tag{5}$$

The solution of the differential equation (3) is

$$n(t) = \frac{n(0)}{1+kn(0)\cdot t} \tag{6}$$

The lifetime of the two-body recombination, i.e., exciton-exciton annihilation, can be defined as

$$\tau_4 = -n_0\left(\frac{dn}{dt}\right)^{-1} = \frac{1}{kn(0)} \propto \frac{1}{kF(0)} \tag{7}$$

where k is the second-order rate constant for recombination, and $n(0)$ is the density of the photoinduced exciton just after the excitation that is proportional to the pump fluence $F(0)$.

Supplementary References

1. Wright AD, Verdi C, Milot RL, Eperon GE, Perez-Osorio MA, Snaith HJ, Giustino F, Johnston MB, Herz LM. Electron-phonon coupling in hybrid lead halide perovskites. *Nat. Commun.* **7**, 11755 (2016).
2. Fang HH, Wang F, Adjokatse S, Zhao N, Even J, Antonietta Loi M. Photoexcitation dynamics in solution-processed formamidinium lead iodide perovskite thin films for solar cell applications. *Light Sci. Appl.* **5**, e16056 (2016).
3. Piana GM, Bailey CG, Lagoudakis PG. Phonon-Assisted Trapping and Re-excitation of Free Carriers and Excitons in Lead Halide Perovskites. *J. Phys. Chem. C* **123**, 19429-19436 (2019).
4. Handa T, Aharen T, Wakamiya A, Kanemitsu Y. Radiative recombination and electron-phonon coupling in lead-free $CH_3NH_3SnI_3$ perovskite thin films. *Phys. Rev. Materials* **2**, 075402 (2018).
5. Ni L, Huynh U, Cheminal A, Thomas TH, Shivanna R, Hinrichsen TF, Ahmad S, Sadhanala A, Rao A. Real-Time Observation of Exciton-Phonon Coupling Dynamics in Self-Assembled Hybrid Perovskite Quantum Wells. *ACS Nano* **11**, 10834-10843 (2017).
6. Wang J, Shen H, Li W, Wang S, Li J, Li D. The Role of Chloride Incorporation in Lead-Free 2D Perovskite $(BA)_2SnI_4$: Morphology, Photoluminescence, Phase Transition, and Charge Transport. *Adv. Sci.* **6**, 1802019 (2019).
7. Leveillee J, Katan C, Even J, Ghosh D, Nie W, Mohite AD, Tretiak S, Schleife A, Neukirch AJ. Tuning Electronic Structure in Layered Hybrid Perovskites with Organic Spacer Substitution. *Nano Lett.* **19**, 8732-8740 (2019).
8. Leveillee J, Katan C, Zhou L, Mohite AD, Even J, Tretiak S, Schleife A, Neukirch AJ. Influence of π-conjugated cations and halogen substitution on the optoelectronic and excitonic properties of layered hybrid perovskites. *Phys. Rev. Materials* **2**, 105406 (2018).
9. Passarelli JV, Fairfield DJ, Sather NA, Hendricks MP, Sai H, Stern CL, Stupp SI. Enhanced Out-of-Plane Conductivity and Photovoltaic Performance in n = 1 Layered Perovskites through Organic Cation Design. *J. Am. Chem. Soc.* **140**, 7313-7323 (2018).
10. Braun M, Tuffentsammer W, Wachtel H, Wolf HC. Tailoring of energy levels in lead chloride based layered perovskites and energy transfer between the organic and inorganic planes. *Chemical Physics Letters* **303**, 157-164 (1999).
11. Ema K, Inomata M, Kato Y, Kunugita H, Era M. Nearly perfect triplet-triplet energy transfer from Wannier excitons to naphthalene in organic-inorganic hybrid quantum-well materials. *Phys. Rev. Lett.* **100**, 257401 (2008).
12. Ishihara T, Takahashi J, Goto T. Exciton-State in Two-Dimensional Perovskite Semiconductor $(C_{10}H_{21}NH_3)_2PbI_4$. *Solid State Commun.* **69**, 933-936 (1989).
13. Xing G, Mathews N, Sun S, Lim SS, Lam YM, Gratzel M, Mhaisalkar S, Sum TC. Long-range balanced electron- and hole-transport lengths in organic-inorganic $CH_3NH_3PbI_3$. *Science* **342**, 344-347 (2013).
14. Elliott RJ. Intensity of Optical Absorption by Excitons. *Phys. Rev.* **108**, 1384-1389 (1957).
15. Pereira MF, Jr. Analytical solutions for the optical absorption of semiconductor superlattices. *Phys. Rev. B* **52**, 1978-1983 (1995).
16. Tanguy C. Complex dielectric constant of two-dimensional Wannier excitons. *Solid State Commun.* **98**, 65-68 (1996).

17. Neutzner S, Thouin F, Cortecchia D, Petrozza A, Silva C, Srimath Kandada AR. Exciton-polaron spectral structures in two-dimensional hybrid lead-halide perovskites. *Phys. Rev. Materials* **2**, 064605 (2018).
18. Sheikh T, Nawale V, Pathoor N, Phadnis C, Chowdhury A, Nag A. Molecular Intercalation and Electronic Two Dimensionality in Layered Hybrid Perovskites. *Angew.Chem. Int. Ed.* **59**, 11653-11659 (2020).
19. Plechinger G, Mann J, Preciado E, Barroso D, Nguyen A, Eroms J, Schuller C, Bartels L, Korn T. A direct comparison of CVD-grown and exfoliated MoS_2 using optical spectroscopy. *Semicond. Sci. Technol.* **29**, 064008 (2014).
20. Odonnell KP, Chen X. Temperature-Dependence of Semiconductor Band-Gaps. *Appl. Phys. Lett.* **58**, 2924-2926 (1991).
21. Fang H-H, Raissa R, Abdu-Aguye M, Adjokatse S, Blake GR, Even J, Loi MA. Photophysics of Organic-Inorganic Hybrid Lead Iodide Perovskite Single Crystals. *Adv. Funct. Mater.* **25**, 2378-2385 (2015).
22. Milot RL, Eperon GE, Snaith HJ, Johnston MB, Herz LM. Temperature-Dependent Charge-Carrier Dynamics in $CH_3NH_3PbI_3$ Perovskite Thin Films. *Adv. Funct. Mater.* **25**, 6218-6227 (2015).
23. D'Innocenzo V, Grancini G, Alcocer MJ, Kandada AR, Stranks SD, Lee MM, Lanzani G, Snaith HJ, Petrozza A. Excitons versus free charges in organo-lead tri-halide perovskites. *Nat. Commun.* **5**, 3586 (2014).
24. Zeches RJ, Rossell MD, Zhang JX, Hatt AJ, He Q, Yang CH, Kumar A, Wang CH, Melville A, Adamo C, Sheng G, Chu YH, Ihlefeld JF, Erni R, Ederer C, Gopalan V, Chen LQ, Schlom DG, Spaldin NA, Martin LW, Ramesh R. A Strain-Driven Morphotropic Phase Boundary in $BiFeO_3$. *Science* **326**, 977-980 (2009).
25. Chen Y, Kothiyal GP, Singh J, Bhattacharya PK. Absorption and Photoluminescence Studies of the Temperature-Dependence of Exciton Life Time in Lattice-Matched and Strained Quantum Well Systems. *Superlattice Microst.* **3**, 657-664 (1987).
26. Saran R, Heuer-Jungemann A, Kanaras AG, Curry RJ. Giant Band gap Renormalization and Exciton Phonon Scattering in Perovskite Nanocrystals. *Adv. Optical Mater.* **5**, 1700231 (2016).
27. Diroll BT. Temperature-Dependent Intraband Relaxation of Hybrid Perovskites. *J. Phys. Chem. Lett.* **10**, 5623-5628 (2019).
28. Wei K, Xu Z, Chen R, Zheng X, Cheng X, Jiang T. Temperature-dependent excitonic photoluminescence excited by two-photon absorption in perovskite $CsPbBr_3$ quantum dots. *Opt. Lett.* **41**, 3821-3824 (2016).
29. Lee J, Koteles ES, Vassell MO. Luminescence linewidths of excitons in GaAs quantum wells below 150 K. *Phys. Rev. B* **33**, 5512-5516 (1986).
30. Ghosh D, Neukirch AJ, Tretiak S. Optoelectronic Properties of Two-Dimensional Bromide Perovskites: Influences of Spacer Cations. *J. Phys. Chem. Lett.* **11**, 2955-2964 (2020).
31. Manser JS, Kamat PV. Band filling with free charge carriers in organometal halide perovskites. *Nat. Photonics* **8**, 737-743 (2014).
32. Muñoz M, Pollak FH, Kahn M, Ritter D, Kronik L, Cohen GM. Burstein-Moss shift of n-doped $In_{0.53}Ga_{0.47}As/InP$. *Phys. Rev. B* **63**, 233302 (2001).
33. Delport G, Chehade G, Ledee F, Diab H, Milesi-Brault C, Trippe-Allard G, Even J, Lauret JS, Deleporte E, Garrot D. Exciton-Exciton Annihilation in Two-Dimensional Halide Perovskites

at Room Temperature. *J. Phys. Chem. Lett.* **10**, 5153-5159 (2019).

34. Yang B, Hong F, Chen JS, Tang YX, Yang L, Sang YB, Xia XS, Guo JW, He HX, Yang SQ, Deng WQ, Han KL. Colloidal Synthesis and Charge-Carrier Dynamics of $Cs_2AgSb_{1-y}Bi_yX_6$ (X: Br, Cl; 0 ≤y ≤1) Double Perovskite Nanocrystals. *Angew.Chem. Int. Ed.* **58**, 2278-2283 (2019).
